# Adaptive fixed-time fault-tolerant trajectory tracking control for disturbed robotic manipulator

**DOI:** 10.1371/journal.pone.0323346

**Published:** 2025-06-25

**Authors:** Zeeshan Anjum, Zhe Sun, Saim Ahmed, Ahmad Taher Azar

**Affiliations:** 1 School of Mechanical and Electrical Engineering, Quanzhou University of Information Engineering, Quanzhou, China; 2 College of Information Engineering, Zhejiang University of Technology, Hangzhou, China; 3 College of Computer and Information Sciences, Prince Sultan University, Riyadh, Saudi Arabia; 4 Automated Systems and Computing Lab (ASCL), Prince Sultan University, Riyadh, Saudi Arabia; Beijing University of Posts and Telecommunications, CHINA

## Abstract

This article introduces a fixed-time trajectory control method for robotic manipulators, aimed at improving trajectory precision despite external disturbances, actuator faults, and uncertainties. Initially, a fast fixed-time nonsingular terminal sliding surface (FFNTSS) is utilized, featuring a bounded convergence time that remains unaffected by the initial conditions. This sliding surface not only prevents the occurrence of singularity but also guarantees fast convergence. Subsequently, building upon the FFNTSS and adaptive methodology, a novel approach termed continuous adaptive fixed-time nonsingular terminal sliding mode fault-tolerant control (CAFNTSMFTC) is introduced. According to the Lyapunov theorem, rigorous analysis demonstrates that the sliding mode variables and tracking errors of the closed-loop system converge to a small neighborhood of the origin within a fixed-time frame. Moreover, by approximating the square of the uncertainty’s upper bound, the devised CAFNTSMFTC approach eliminates the need for the boundary layer commonly imposed in existing adaptive fixed-time control approaches. Lastly, comprehensive comparative simulations are conducted employing the PUMA560 robot. These simulations validate the proposed control strategy, underscoring its ability to achieve precise trajectory tracking and fast convergence, even when facing uncertainties, disturbances, and actuator faults. Moreover, the proposed control strategy for the robot manipulator is distinguished by its continuity and demonstrates dynamics in which chattering is mitigated.

## 1 Introduction

Robot manipulators have found ongoing use in manufacturing settings, primarily to enhance operational speed and overall throughput. Their application in manufacturing is extensive, encompassing tasks such as pick-and-place operations, machining, material handling, inspection and testing, assembly and welding [1]. The precise tracking of trajectories by the manipulator system is essential for all the tasks listed above. Broadly speaking, the ability of robot manipulators to accurately follow trajectories is primarily influenced by two main factors: firstly, the intricate dynamics, and secondly, the effectiveness of the actuators, including potential faults. As a result, developing a robust and fault-tolerant tracking control strategy for robot manipulators remains a challenging endeavor [2,3].

Owing to the interconnected mechanical characteristics of robot manipulators, various uncertainties such as reduced input voltage and increased load have been influencing the tracking performance within robot system applications. In response to these challenges, numerous fault-tolerant control (FTC) strategies have been devised in [4] to ensure both reliability and tracking performance. In general, the ability of a system to withstand faults and failures can be improved by employing either redundant hardware setups or robust controllers. While the former approach adds complexity to the system design [6,7], the latter is more suitable for real-world applications due to its simpler design [8,9]. Extensive efforts have been dedicated to the advancement of FTC design, aiming to effectively manage the consequences of faults and enhance the overall robustness and resilience of systems. Typically, FTC strategies can be crafted employing either an active or passive methodology. Within the active FTC framework, fault information acquired through feedback from a fault diagnosis observer is harnessed to modify the standard controller, as detailed in reference [10]. The downside of this strategy lies in its necessity for an extra fault diagnosis observer, contributing to a delay in fault compensation timing. In contrast, the passive FTC capitalizes on the controller’s robust characteristics to manage the consequences of faults within the systems [11]. Furthermore, it is a common practice to treat actuator faults in passive FTC systems as supplementary disturbances [12]. While the passive FTC approach exhibits quicker responses for compensating actuator faults compared to active FTC, it’s noteworthy that the passive FTC system demands greater control inputs than its active counterpart [12]. Consequently, there arises a need to formulate a fault-tolerant tracking control strategy that not only boasts heightened robustness and a straightforward structure but also addresses these disparities. This strategy is aimed at simultaneously mitigating the impact of uncertainties and actuator faults on tracking performance. With the aim of enhancing the tracking performance of robot manipulators when confronted with uncertain dynamics, external disturbances, and partial loss of actuator effectiveness faults, numerous methodologies have emerged. Initial approaches encompass PID control [13], intelligent and learning controls [5,14], optimal controls [15], and robust controls [16]. Among these, robust controls stand out for their elevated robustness and proficiency in mitigating disturbances and/or faults. Because of its inherent robustness, sliding mode control (SMC) has found widespread application in the realm of FTC for robot manipulators [17,18]. To bolster the system’s robustness, an SMC of third-order has been formulated in [4]. Likewise, in reference [19], an integral sliding mode control (ISMC) has been developed with the identical goal. Nonetheless, a limitation of these controllers is their inability to guarantee the system’s convergence within a finite time frame.

In order to attain convergence of the system’s tracking error signals within a finite time frame, researchers have formulated and introduced finite-time fault-tolerant controllers. In reference [20], a finite time FTC approach has been developed for robot manipulator. This method incorporates nonsingular fast terminal SMC and employs time delay estimation technique. In reference [21], an improved and resilient FTC method is introduced for robot manipulators. This technique combines nonsingular fast terminal SMC with adaptive fuzzy PID approach. In reference [12], a novel approach to nonsingular fast terminal SMC is crafted using an adaptive backstepping technique to enhance fault tolerance in robotic manipulator. Additionally, by incorporating the backstepping control strategy, FTC methods have been put forth for a specific set of nonlinear systems in [22,23]. These techniques contribute to achieving superior tracking performance with convergence within a finite time period. A slight limitation of finite-time controls pertains to their comparatively slower convergence in contrast to exponentially stable systems, especially when the system’s states are far from the equilibrium position. This phenomenon can be attributed to the fact that the time it takes for finite-time controls to settle varies based on the initial states of closed-loop systems. Consequently, the convergence performance differs across the closed-loop system’s various initial states. To address this limitation, researchers have explored fixed-time controllers [24,25]. The advantages of fixed-time controllers lie in their ability to achieve a fixed convergence time that is unaffected by initial states. This approach allows for the determination of the maximum system convergence time during controller design, offering valuable advance insights into system performance. A specific variant known as fixed-time SMC has undergone extensive development to achieve both fixed-time convergence and improved robustness [26,27]. It’s worth noting that when employing a fixed-time terminal sliding mode, the current approach might encounter the singularity phenomenon. To address this issue and avoid singularity, several alternative control strategies involving the use of fixed-time nonsingular terminal sliding mode surfaces have been suggested in previous studies [28,29]. However, these prior methods demand substantial switching gains to mitigate uncertainties, which unfortunately result in an undesirable chattering phenomenon.To address this issue, adaptive controllers have been designed to effectively minimize chattering [30–32]. In reference [33], an adaptive approach is incorporated into the fixed-time nonsingular terminal sliding mode control (FTNTSMC) technique in order to mitigate chattering. This involves dynamically tuning the switching gains to estimate the maximum limits of uncertainties. Despite these efforts, the chattering phenomenon remains unavoidable due to the presence of the sign function within these control methods. According to the authors’ comprehension, the task of developing an FTNTSMC method free from a sign function, with the aim of entirely eliminating chattering, remains an unresolved issue when dealing with trajectory tracking control for robot manipulators amidst uncertainties,external disturbances, and potential actuator faults.

Motivated by the insights mentioned earlier, a new trajectory tracking controller with fixed-time characteristics is formulated for the robot manipulator in the presence of uncertainties, external disturbances, and actuators fault. The primary advancements of this study can be outlined as follows:

- A novel method for adaptive, fault-tolerant trajectory tracking control in robotic manipulators is proposed. This approach incorporates a FFNTSS that ensures convergence within a bounded time, independent of initial conditions. An estimation of the upper bound of the settling time is also provided. The proposed FFNTSS effectively addresses the singularity problem while offering improved convergence performance.
- Differing from the adaptive fixed-time controllers mentioned in prior works [34,35], this research presents a novel adaptive law to formulate a continuous adaptive FNTSM fault-tolerant control CAFNTSMFTC. The proposed controller ensures fixed-time tracking performance despite the presence of external disturbances, actuator faults, and uncertainties, while also effectively reducing the chattering phenomenon.
- Furthermore, in accordance with the Lyapunov theorem, rigorous analysis is performed to illustrate that the position tracking errors of the closed-loop system converge to a narrow region around zero within a fixed-time frame.

The remaining portions of this work follow the following structure: The subsequent section introduces the relevant preliminary information and define the problem. Sect 3 showcases the main results, highlighting an adaptive fixed-time fault-tolerant trajectory tracking control system for robot manipulators that attains highly precise trajectory tracking control within a fixed time frame. Sect 4 features the presentation of verification through simulation experiments. Lastly, Sect 5 provides the conclusion.

## 2 Problem description and mathematical foundations

### 2.1. Problem description

**Fig 1:**
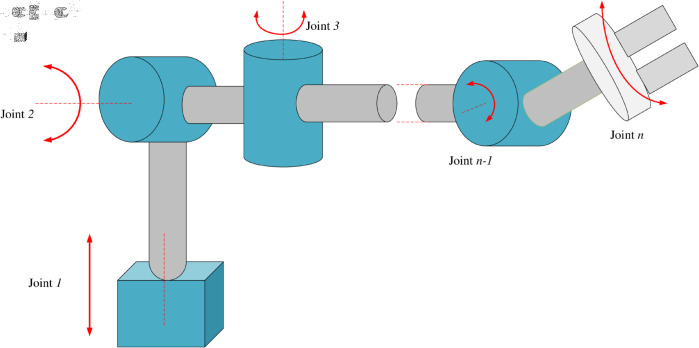
n-DOF rigid manipulator.

The dynamic equation in joint space for an *n*- degree of freedom rigid manipulator, as illustrated in Fig 1, can be formulated by considering uncertainties, disturbances, and actuator faults as provided in [12]:

$${\ddot{q}}_{\iota }={𝐌}^{-1}({𝐪}_{\iota })(\tau -{𝐙}_{\iota }({𝐪}_{\iota },{\dot{q}}_{\iota }){\dot{q}}_{\iota }-{𝐆}_{\iota }({𝐪}_{\iota })-{𝐥}_{d}-{𝐅}_{\iota }({\dot{q}}_{\iota }))+\Omega_{1}(t-T_{af})\Omega_{2}({𝐪}_{\iota },{\dot{q}}_{\iota },\tau )$$ (1)

where the symbol $𝐌({𝐪}_{\iota })\in {ℜ}^{n\times n}$ represents the inertia matrix, which is always positive and definite. ${𝐪}_{\iota }\in {ℜ}^{n}$ denotes the position vector, while ${\dot{q}}_{\iota }\in {ℜ}^{n}$ and ${\ddot{q}}_{\iota }\in {ℜ}^{n}$ represent the velocity and acceleration vectors, respectively. $\tau \in {ℜ}^{n}$ stands for the actuator inputs applied to the system. The matrix ${𝐙}_{\iota }({𝐪}_{\iota },{\dot{q}}_{\iota })\in {ℜ}^{n\times n}$ encompasses centripetal and Coriolis forces. ${𝐆}_{\iota }({𝐪}_{\iota })\;\in \;{ℜ}^{n}$ represents the gravity vector. ${𝐅}_{\iota }({\dot{q}}_{\iota })\in {ℜ}^{n}$ signifies the friction matrix, and ${𝐥}_{d}\in {ℜ}^{n}$ indicates the load disturbance matrix. $\Omega_{2}({𝐪}_{\iota },{\dot{q}}_{\iota },\tau )\in {ℜ}^{n}$ is used to characterize the system’s response to faults, $\Omega_{1}(t$ − *T*_*af*_) depicts the time profile of these faults, and *T*_*af*_ represents the time at which these faults occur.

In the realm of robot dynamics, the $\Omega_{1}(t-T_{af})$ is typically depicted as a diagonal matrix, taking the following form:

$$\Omega_{1}(t-T_{af})=diag\{\Omega_{11}(t-T_{af1}),\Omega_{12}(t-T_{af2}),...,\Omega_{1n}(t-T_{afn})\}$$ (2)

where $\Omega_{1i}$ characterizes the impact of the fault on the *i*th state equation, $T_{afi},\;i=1,\;2,....,n$ represent the time instances at which faults occur in the *i*th joint. It has been established that the fault’s time profile exhibits the following pattern:

$$\Omega_{1i}\left(t-T_{afi}\right)=\{0\;\;\;\;\;\;\;\;\;\;\;\;\;\;\;\;\;\;\;\;\;\;\;\;\;\;\;\;\;\;\;\;\;\;\;if\;t\;<\;T_{afi}\;1-e^{-\Gamma_{1i}}\left(t-T_{afi}\right)\;\;\;\;\;\;if\;t\geq T_{afi}\;\;$$ (3)

$\Gamma_{1i}$ represents the rate at which unidentified defects progress. A low value of $\Gamma_{1i}$ suggests that the fault is evolving slowly, categorizing it as an incipient fault. Conversely, a high value of $\Gamma_{1i}$ implies that $\Omega_{1i}$ has transitioned into a step function, classifying the fault as an abrupt fault.

In the realm of robotics, actuator and sensor failures are frequent occurrences. This research delves into the repercussions of actuator faults within the system, as outlined in reference [36]. Specifically, the focus is on gain faults and bias faults. When either of the mentioned faults arises, the control signal described in Eq (1) can be expressed as follows:

$$\tau_{f}=\partial_{1}\tau +\partial_{2}\tau$$ (4)

where $\partial_{1}\in {ℜ}^{n}$ represents the gain fault and $\partial_{2}\in {ℜ}^{n}$ denotes the bias fault, $\tau_{f}$ is used to represent the actual value and τ is used to denote the desired value of torque. Under these circumstances, the fault function $\Omega_{2}({𝐪}_{\iota },{\dot{q}}_{\iota },\tau )\in {ℜ}^{n}$ in Eq (1) can be reformulated as follows:

$$\Omega_{2}({𝐪}_{\iota },{\dot{q}}_{\iota },\tau )={𝐌}^{-1}({𝐪}_{\iota })\left(\partial_{1}-I\right)\tau +{𝐌}^{-1}({𝐪}_{\iota })\partial_{2}\tau .$$ (5)

The dynamic model (1) can be further expressed in the following form:

$${\ddot{q}}_{\iota }={𝐌}^{-1}({𝐪}_{\iota })\tau +{𝐐}_{\iota }({𝐪}_{\iota },{\dot{q}}_{\iota })+{𝐃}_{\iota }$$ (6)

here, ${𝐐}_{\iota }({𝐪}_{\iota },{\dot{q}}_{\iota })={𝐌}^{-1}({𝐪}_{\iota })(-{𝐙}_{\iota }({𝐪}_{\iota },{\dot{q}}_{\iota }){\dot{q}}_{\iota }$ − ${𝐆}_{\iota }({𝐪}_{\iota }))$ symbolizes the known lumped component of the robotic manipulator’s dynamics, while ${𝐃}_{\iota }={𝐌}^{-1}({𝐪}_{\iota })(-{𝐥}_{d}$ − ${𝐅}_{\iota }({\dot{q}}_{\iota }))$  +  $\Omega_{1}(t$ − $T_{af})\Omega_{2}({𝐪}_{\iota },{\dot{q}}_{\iota },\tau )$ represents the lumped component of the robotic manipulator’s dynamics that remains unknown.

We can write the Eq (6) in state-space form as follows if we define the state variables to be ${ℵ}_{1}={𝐪}_{\iota }$ and ${ℵ}_{2}={\dot{q}}_{\iota }$ respectively:

$$\{{\dot{ℵ}}_{1}={ℵ}_{2}\;{\dot{ℵ}}_{2}={𝐌}^{-1}({ℵ}_{1})\tau +{𝐐}_{\iota }({ℵ}_{1},{ℵ}_{2})+{𝐃}_{\iota }\;$$ (7)

By utilizing Eq (7) and introducing ${\tilde{q}}_{1}={ℵ}_{1}$ − ${𝐪}_{d}$, ${\tilde{q}}_{2}={ℵ}_{2}$ − ${\dot{q}}_{d}$ where ${𝐪}_{d}\in {ℜ}^{n}$ represents the desired trajectory, and ${\dot{q}}_{d}\in {ℜ}^{n}$ represents the derivative of the desired trajectory, we can express the error equation of the robotic manipulator in the following manner:

$$\{{\dot{\tilde{q}}}_{1}={\tilde{q}}_{2}\;{\dot{\tilde{q}}}_{2}={𝐂}_{\iota }(\tilde{q})𝐮+{𝐇}_{\iota }(\tilde{q})+{𝐃}_{\iota }\;$$ (8)

with ${𝐂}_{\iota }(\tilde{q})={𝐌}^{-1}({ℵ}_{1}),\;{𝐇}_{\iota }(\tilde{q})={𝐐}_{\iota }({ℵ}_{1},{ℵ}_{2})-{\ddot{q}}_{d},$ and 𝐮=τ.

### 2.2. Mathematical foundations

Consider the following system

$$\dot{y}=f\left(y\left(t\right)\right),y\left(0\right)=y_{0},f\left(0\right)=0$$ (9)

where $y\in {ℜ}^{n}$ and $f:\;{ℜ}^{n}\to {ℜ}^{n}$ denotes a nonlinear function.

**Definition 1.** [37] System (9) is classified as fixed-time stable when it exhibits global finite-time stability. Consequently, it will reach the origin within a bounded convergence time $T\left(y_{0}\right)$. In this context, there exists a positive constant $T_{\max}$ such $T\left(y_{0}\right)\;<\;T_{\max}$.

**Lemma 1.** [24] Assume that for system (9), there exists a Lyapunov function V(x), with parameters $\alpha_{1},\;\beta_{1},\;p,\;q,\;k\in {ℜ}^{+}$, 0 < *p* < 1 and *q* > 1, such that the inequality $\dot{V}\left(y\right)\leq -\left(\alpha_{1}V{\left(y\right)}^{p}+\beta_{1}V{\left(y\right)}^{q}\right)$ holds. The system is then fixed time stable. In addition, the upper bound of the convergence time is given below.

$$T\leq \frac{1}{\alpha_{1}\left(1-p\right)}+\frac{1}{\beta_{1}\left(q-1\right)}$$ (10)

**Lemma 2.** [37] For any $y_{i}\in ℜ,\;i=1,2,3,...,n$ if $v\in {ℜ}^{+}$ and 0<v≤1, we have $\sum {\mathrm{\backslash nolimits}}_{i=1}^{n}{\left|y_{i}\right|}^{v}\geq {\left(\sum {\mathrm{\backslash nolimits}}_{i=1}^{n}\left|y_{i}\right|\right)}^{v}$.

**Lemma 3.** [37] For any $y_{i}\in ℜ,\;i=1,2,3,...,n$, and $\sum {\mathrm{\backslash nolimits}}_{i=1}^{n}{\left|y_{i}\right|}^{v_{1}}\geq n^{1-v_{1}}{\left(\sum {\mathrm{\backslash nolimits}}_{i=1}^{n}\left|y_{i}\right|\right)}^{v_{1}}$ where $v_{1}\in {ℜ}^{+}$ and $v_{1}\;>\;1$.

**Lemma 4.** [38] The inequality $y_{2}^{d}-y_{1}^{d}\leq {\left(y_{1}-y_{2}\right)}^{d}$, holds true with $d\;>\;1,\;y_{1}\;>\;0$ and $y_{1}\geq y_{2}$.

**Lemma 5.** [38] If $0\;<\;\bar{v},$ $y_{1}\geq 0,\;y_{2}\;>\;0$ we have

$$\frac{1}{\bar{v}+1}\left(y_{2}^{\bar{v}+1}-y_{1}^{\bar{v}+1}\right)\geq \left(y_{2}-y_{1}\right)y_{1}^{\bar{v}}.$$ (11)

**Notations**: For a vector $𝐲={\left[y_{1},y_{2},....,y_{n}\right]}^{\text{T}},$ we can have $\;sig{\left(𝐲\right)}^{\Theta }=[\text{si}{\text{g}}^{\Theta }\left(y_{1}\right),\;\text{si}{\text{g}}^{\Theta }\left(y_{2}\right),...$ $\text{si}{\text{g}}^{\Theta }\left(y_{n}\right){]}^{\text{T}}$ with $\text{si}{\text{g}}^{\Theta }\left(y_{i}\right)={\left|y_{i}\right|}^{\Theta }\text{sign}\left(y_{i}\right),$ i=1,2,3,...,n, Θ∈ℜ is a constant and sign(.) is denoting the sign function. |•| represents the absolute value of a scalar.

## 3 Adaptive fixed-time nonsingular trajectory tracking control design

**Fig 2:**
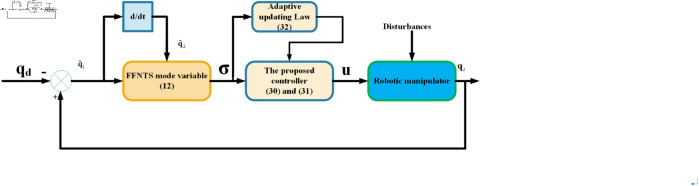
Schematic illustration of the control system.

In this segment, the primary objective is to enhance the trajectory tracking performance of a robot manipulator in the presence of uncertainties, external disturbances and actuator faults while achieving fast convergence of the tracking error. To attain this goal, the focus is placed on creating an innovative CAFNTSMFTC method. Additionally, the fixed-time stability of the closed-loop system is formally verified and supported through theoretical evidence. Fig 2 depicts the overall structural block diagram for the control mechanism suggested in this paper.

### 3.1. FFNTSS

Within this particular subsection, with the objective of preventing the singularity problem, a FFNTS mode variable is initially implemented as follows:

$$\sigma ={\tilde{q}}_{2}+\alpha_{1}sig{\left({\tilde{q}}_{1}\right)}^{\frac{\Theta_{1}}{\Theta_{2}}}+\alpha_{2}\sigma_{d}\left({\tilde{q}}_{1}\right)+\alpha_{3}{\tilde{q}}_{1}$$ (12)

where $\alpha_{1},\;\alpha_{2}$ and $\alpha_{3}$ are the symbols to denote the diagonal matrices consist of diagonal elements $\alpha_{1i},\;\alpha_{2i}$ and $\alpha_{3i}$ with values greater than zero and $\Theta_{1}$ and $\Theta_{2}$ denote positive integers which satisfy $\Theta_{1}$ > $\Theta_{2}$ . Moreover the *i*th term of $\sigma_{d}\left({\tilde{q}}_{1}\right)$ can be stated as follows:

$$\sigma_{d}{\left({\tilde{q}}_{1i}\right)}_{i}=\{\text{si}{\text{g}}^{\frac{\Theta_{3}}{\Theta_{4}}}\left({\tilde{q}}_{1i}\right)\;\;\;if\;{\bar{\sigma }}_{i}=0\;\;\text{or}\;\left({\bar{\sigma }}_{i}\neq 0,\;\left|{\tilde{q}}_{1i}\right|\geq \Delta_{i}\right)\;\;j_{1i}{\tilde{q}}_{1i}+j_{2i}\text{sign}\left({\tilde{q}}_{1i}\right){\tilde{q}}_{1i}^{2}\;\;\;\;if\;{\bar{\sigma }}_{i}\neq 0,\;\left|{\tilde{q}}_{1i}\right|\;<\;\Delta_{i}\;\;\;\;\;\;$$ (13)

where $\Theta_{3}$ and $\Theta_{4}$ denote positive integers which satisfy $\Theta_{3}$ < $\Theta_{4}$, and *j*_1*i*_ and *j*_2*i*_ are formulated in a form $j_{1i}=\left(2\Theta_{4}-\Theta_{3}/\Theta_{4}\right)\Delta_{i}^{\left(\Theta_{3}/\Theta_{4}\right)-1}$ and $j_{2i}=\left(\Theta_{3}-\Theta_{4}/\Theta_{4}\right)\Delta_{i}^{\left(\Theta_{3}/\Theta_{4}\right)-2}$ respectively, to guarantee the continuity of the sliding mode surface (12). $\Delta_{i}$ represents a positive constant. Furthermore, ${\bar{\sigma }}_{i}$ in the above (13) is used to show the term stated below

$${\bar{\sigma }}_{i}={\tilde{q}}_{2i}+\alpha_{1i}\text{si}{\text{g}}^{\frac{\Theta_{1}}{\Theta_{2}}}\left({\tilde{q}}_{1i}\right)+\alpha_{2i}\text{si}{\text{g}}^{\frac{\Theta_{3}}{\Theta_{4}}}\left({\tilde{q}}_{1i}\right)+\alpha_{3i}{\tilde{q}}_{1i}$$ (14)

**Theorem 1.** Take into account the error dynamic model described by (8), once its states reach the FFNTSS, i.e., $\sigma_{i}={\bar{\sigma }}_{i}=0$ then ${\tilde{q}}_{1i}=0$ and ${\tilde{q}}_{2i}=0$ can be attained in fixed-time regardless of the initial values of the system states. Furthermore, the duration required for settling can be expressed as follows:

$$T_{s}\leq \frac{2\Theta_{4}}{\alpha_{6i}\left(\Theta_{4}-\Theta_{3}\right)}\text{ln}\left(1+\alpha_{6i}/\alpha_{5i}\right)+\frac{2\Theta_{2}}{\alpha_{4i}\left(\Theta_{1}-\Theta_{2}\right)}$$ (15)

*Proof:* To underscore the straightforward nature of this demonstration, the analysis focuses on the *i*th degree of freedom (DOF) of the rigid manipulator. Once the condition $\sigma_{i}={\bar{\sigma }}_{i}=0$ is achieved, it becomes possible to obtain that

$${\tilde{q}}_{2i}=-\left(\alpha_{1i}\text{si}{\text{g}}^{\frac{\Theta_{1}}{\Theta_{2}}}\left({\tilde{q}}_{1i}\right)+\alpha_{2i}\text{si}{\text{g}}^{\frac{\Theta_{3}}{\Theta_{4}}}\left({\tilde{q}}_{1i}\right)+\alpha_{3i}{\tilde{q}}_{1i}\right)$$ (16)

By employing a Lyapunov function as given below we have

$$V_{1i}=0.5{\tilde{q}}_{1i}^{2}$$ (17)

Upon computing the time derivative of the equation mentioned above, we obtain

$${\dot{V}}_{1i}={\tilde{q}}_{1i}{\tilde{q}}_{2i}$$ (18)

Now, by substituting Eq (16) into the aforementioned equation, we arrive at

$${\dot{V}}_{1i}=-{\tilde{q}}_{1i}\left(\alpha_{1i}\text{si}{\text{g}}^{\frac{\Theta_{1}}{\Theta_{2}}}\left({\tilde{q}}_{1i}\right)+\alpha_{2i}\text{si}{\text{g}}^{\frac{\Theta_{3}}{\Theta_{4}}}\left({\tilde{q}}_{1i}\right)+\alpha_{3i}{\tilde{q}}_{1i}\right)\;\;\;\;\;\;\;=-\alpha_{1i}{\left|{\tilde{q}}_{1i}\right|}^{\frac{\Theta_{1}+\Theta_{2}}{\Theta_{2}}}-\alpha_{2i}{\left|{\tilde{q}}_{1i}\right|}^{\frac{\Theta_{3}+\Theta_{4}}{\Theta_{4}}}-\alpha_{3i}{\tilde{q}}_{1i}^{2}\;\;\;\;\;\;\;=-\alpha_{1i}2^{\frac{\Theta_{1}+\Theta_{2}}{2\Theta_{2}}}V_{1i}^{\frac{\Theta_{1}+\Theta_{2}}{2\Theta_{2}}}-\alpha_{2i}2^{\frac{\Theta_{3}+\Theta_{4}}{2\Theta_{4}}}V_{1i}^{\frac{\Theta_{3}+\Theta_{4}}{2\Theta_{4}}}-2\alpha_{3i}V_{1i}\;\;\;\;\;\;\;=-\alpha_{4i}V_{1i}^{\frac{\Theta_{1}+\Theta_{2}}{2\Theta_{2}}}-\alpha_{5i}V_{1i}^{\frac{\Theta_{3}+\Theta_{4}}{2\Theta_{4}}}-\alpha_{6i}V_{1i}\;\;$$ (19)

where $\alpha_{4i}=\alpha_{1i}2^{\frac{\Theta_{1}+\Theta_{2}}{2\Theta_{2}}},\;\alpha_{5i}=\alpha_{2i}2^{\frac{\Theta_{3}+\Theta_{4}}{2\Theta_{4}}},$ and $\alpha_{6i}=2\alpha_{3i}$. Now if $\left|{\tilde{q}}_{1i}\right|>1$, we can deduce from above equation as given below

$${\dot{V}}_{1i}\leq -\alpha_{4i}V_{1i}^{\frac{\Theta_{1}+\Theta_{2}}{2\Theta_{2}}}$$ (20)

And in contrast to above (20) when $\left|{\tilde{q}}_{1i}\right|\leq 1$ we have

$${\dot{V}}_{1i}\leq -\alpha_{5i}V_{1i}^{\frac{\Theta_{3}+\Theta_{4}}{2\Theta_{4}}}-\alpha_{6i}V_{1i}$$ (21)

Introducing a new term $x=V_{1i}^{\frac{\Theta_{4}-\Theta_{3}}{2\Theta_{4}}},$ the inequality in (20) can be stated as follows

$$\dot{x}\leq -\alpha_{4i}\left(\frac{\Theta_{4}-\Theta_{3}}{2\Theta_{4}}\right)x^{\ell }$$ (22)

where $\ell =\left(\Theta_{4}\Theta_{1}-\Theta_{2}\Theta_{3}\right)/\left(\Theta_{2}\left(\Theta_{4}-\Theta_{3}\right)\right)\;,$ and the inequality in (21) can be stated as

$$\dot{x}\leq -\left(\frac{\Theta_{4}-\Theta_{3}}{2\Theta_{4}}\right)\left(\alpha_{6i}x+\alpha_{5i}\right)$$ (23)

As a result, one may calculate the upper bound of the settling time by solving differential Eqs 22 and (23).

$$T_{s}\leq \underset{x_{0}\to \infty }{\text{lim}}\;\frac{2\Theta_{4}}{\left(\Theta_{4}-\Theta_{3}\right)}\left[\int_{0}^{1}\frac{1}{\alpha_{6i}x+\alpha_{5i}}dx+\int_{0}^{x_{0}}\frac{1}{\alpha_{4i}x^{\ell }}dx\right]\;\;\;\;\;\leq \underset{x_{0}\to \infty }{\text{lim}}\;\frac{2\Theta_{4}}{\left(\Theta_{4}-\Theta_{3}\right)}\left[\frac{1}{\alpha_{6i}}\text{ln}\left(1+\frac{\alpha_{6i}}{\alpha_{5i}}\right)+\frac{\left(x_{0}^{1-\ell }-1\right)}{\alpha_{4i}\left(1-\ell \right)}\right]\;\;\;\;\;\;\leq \frac{2\Theta_{4}}{\left(\Theta_{4}-\Theta_{3}\right)}\left[\frac{1}{\alpha_{6i}}\text{ln}\left(1+\frac{\alpha_{6i}}{\alpha_{5i}}\right)+\frac{1}{\alpha_{4i}\left(\ell -1\right)}\right]\;\;\;\;\;\leq \frac{2\Theta_{4}}{\alpha_{6i}\left(\Theta_{4}-\Theta_{3}\right)}\text{ln}\left(1+\frac{\alpha_{6i}}{\alpha_{5i}}\right)+\frac{2\Theta_{2}}{\alpha_{4i}\left(\Theta_{1}-\Theta_{2}\right)}$$ (24)

The settling time (24) is less than the settling time described in the study conducted by [39].

### 3.2. Design of CAFNTSMFTC law

The dynamics of the FFNTSS are derived by taking into account Eq (12) and the dynamic model of the error as given in Eq (8).

$$\dot{\sigma }={\dot{\tilde{q}}}_{2}+\alpha_{1}\frac{\Theta_{1}}{\Theta_{2}}diag\left({\left|{\tilde{q}}_{1i}\right|}^{\frac{\Theta_{1}}{\Theta_{2}}-1}\right){\tilde{q}}_{2}+\alpha_{2}{\dot{\sigma }}_{d}\left({\tilde{q}}_{1}\right)+\alpha_{3}{\tilde{q}}_{2}\;\;\;\;={𝐁}_{1}+{𝐂}_{\iota }(\tilde{q})(𝐮)+{𝐃}_{\iota }+\alpha_{2}{\dot{\sigma }}_{d}\left({\tilde{q}}_{1}\right)\;$$ (25)

where ${𝐁}_{1}={𝐇}_{\iota }(\tilde{q})+\left(\alpha_{1}\frac{\Theta_{1}}{\Theta_{2}}diag\left({\left|{\tilde{q}}_{1i}\right|}^{\frac{\Theta_{1}}{\Theta_{2}}-1}\right)+\alpha_{3}\right){\tilde{q}}_{2}$. Moreover, ${\dot{\sigma }}_{d}{\left({\tilde{q}}_{1i}\right)}_{i}$ can be represented as stated below

$${\dot{\sigma }}_{d}{\left({\tilde{q}}_{1i}\right)}_{i}=\{\frac{\Theta_{3}}{\Theta_{4}}{\left|{\tilde{q}}_{1i}\right|}^{\frac{\Theta_{3}}{\Theta_{4}}-1}{\tilde{q}}_{2i}\;\;\;\;\;\;\;\;\;\;\;if\;{\bar{\sigma }}_{i}=0\;\;\text{or}\;{\bar{\sigma }}_{i}\neq 0,\;\;\left|{\tilde{q}}_{1i}\right|\geq \Delta_{i}\;j_{1i}{\tilde{q}}_{2i}+2j_{2i}\left|{\tilde{q}}_{1i}\right|{\tilde{q}}_{2i}\;\;\;\;if\;{\bar{\sigma }}_{i}\neq 0,\;\left|{\tilde{q}}_{1i}\right|\;<\;\Delta_{i}\;\;\;\;\;\;$$ (26)

Due to physical limitations and finite energy constraints, external disturbances and actuator faults (such as partial loss of effectiveness or bias faults) are naturally bounded in real-world robotic systems.For instance, actuators are not capable of producing infinite forces or torques, and external disturbances are inherently constrained by the operating conditions of the system. Consequently, it follows that the *i*th term of ${𝐃}_{\iota }$ satisfies:

$$\left|D_{\iota i}\right|\leq b_{0i}+b_{1i}\left|q_{\iota i}\right|+b_{2i}{\left|{\dot{q}}_{\iota i}\right|}^{2}$$ (27)

where $b_{0i},\;b_{1i},\;b_{2i}$ denote positive constants. This structure explicitly accounts for the dependence of disturbances and faults on the joint position $q_{\iota i}$ and velocity ${\dot{q}}_{\iota i}$ which are essential to the manipulator’s dynamic behavior. Moreover, this bound can be simplified to:

$$\left|D_{\iota i}\right|\;\leq a_{1i}+a_{2i}G_{i}$$ (28)

where $a_{0i}=b_{0i}$ and $a_{2i}=\max\left\{b_{1i},b_{2i}\right\}$ denote positive constant which are not known, *G*_*i*_  =  $\left|q_{\iota i}\right|$  +  ${\left|{\dot{q}}_{\iota i}\right|}^{2}$. In control theory, such assumptions are widely accepted and supported by practical observations [40]. Subsequently, the subsequent inequality is derived as follows:

$${\left|D_{\iota i}\right|}^{2}\leq \left(a_{1i}+a_{2i}G_{i}\right)\left(a_{1i}+a_{2i}G_{i}\right)\;\;\;\;\;\;\;\;\;\;\;=\varpi_{1i}+\varpi_{2i}G_{i}+\varpi_{3i}G_{i}^{2}\;$$ (29)

where $\varpi_{1i}=a_{1i}^{2},\;\varpi_{2i}=2a_{1i}a_{2i},$ and $\varpi_{3i}=a_{2i}^{2}$ are positive constant which are not known.

Based on the preceding analysis, a CAFNTSMFTC approach is set to be created for the purpose of tracking robot manipulator trajectories, as outlined below:

$$𝐮=-{𝐂}_{\iota }{\left(\tilde{q}\right)}^{-1}\left({𝐁}_{1}+\alpha_{2}{\dot{\sigma }}_{d}\left({\tilde{q}}_{1}\right)+r_{1}sig{\left(\sigma \right)}^{\gamma_{1}}+r_{2}sig{\left(\sigma \right)}^{\gamma_{2}}+r_{3}\sigma +{𝐔}_{adp}\right)$$ (30)

where $r_{1},\;r_{2},\;r_{3},\;\gamma_{1}\;\;$and $\gamma_{2}$ all are used to represent the matrices of the parameters satisfying $r_{1i}>0,\;r_{2i}>0,\;r_{3i}>0,\;0<\gamma_{1i}<1\;\;$ and $\gamma_{2i}>1.$ $𝐂(\tilde{q})$ is defined in (8) and ${𝐁}_{1}$ is expressed in Eq (25). Moreover considering the practical difficulty of obtaining advance information regarding lumped disturbances, which encompass actuator faults and external disturbances, this paper utilizes an adaptive methodology, to estimate their unidentified parameters. The expression for the *i*th term of ${𝐔}_{adp}$ can be articulated as follows:

$${\left(U_{adp}\right)}_{i}=\frac{\sigma_{i}}{2\nu_{1i}^{2}}{\hat{\varpi }}_{1i}+\frac{\sigma_{i}}{2\nu_{1i}^{2}}{\hat{\varpi }}_{2i}G_{i}+\frac{\sigma_{i}}{2\nu_{1i}^{2}}{\hat{\varpi }}_{3i}G_{i}^{2}$$ (31)

The laws governing the update of adaptations are defined as follows:

$${\dot{\hat{\varpi }}}_{1i}=-\nu_{2i}{\hat{\varpi }}_{1i}^{\gamma_{2i}}-\nu_{5i}{\hat{\varpi }}_{1i}^{\gamma_{1i}}+\frac{1}{2\nu_{1i}^{2}}\eta_{1i}|\sigma_{i}{|}^{2}\;{\dot{\hat{\varpi }}}_{2i}=-\nu_{3i}{\hat{\varpi }}_{2i}^{\gamma_{2i}}-\nu_{6i}{\hat{\varpi }}_{2i}^{\gamma_{1i}}+\frac{1}{2\nu_{1i}^{2}}\eta_{2i}|\sigma_{i}{|}^{2}G_{i}\;{\dot{\hat{\varpi }}}_{3i}=-\nu_{4i}{\hat{\varpi }}_{3i}^{\gamma_{2i}}-\nu_{7i}{\hat{\varpi }}_{3i}^{\gamma_{1i}}+\frac{1}{2\nu_{1i}^{2}}\eta_{3i}|\sigma_{i}{|}^{2}G_{i}^{2}\;$$ (32)

where $\eta_{ji}\;\left(j=1,\;2,\;3\right),\nu_{ki}\;\left(k=1,\;2,\;3,\;4,\;5,\;6,\;7\right)$ are used to express positive real numbers. ${\hat{\varpi }}_{1i},\;{\hat{\varpi }}_{2i}$ and ${\hat{\varpi }}_{3i}$ are used to express the estimation of the $\varpi_{1i},\;\varpi_{2i}$ and $\varpi_{3i}$. Additionally, the initial conditions are selected such that ${\hat{\varpi }}_{ji}\left(0\right)$ > 0. As indicated in [41], it is evident that for all *t* > 0, ${\hat{\varpi }}_{ji}\left(t\right)$ remains greater than 0 when the initial values are greater than zero.

**Remark 1.** As evidenced by equations in (32), the constructed adaptive update laws are rooted in a fractional state feedback framework, distinguishing them from conventional linear feedback techniques. This update law design draws its inspiration from the concept of a power rate reaching law introduced in [42]. Consequently, the suggested adaptive update laws have the potential to expedite the adaptive rate of estimation for parameters $\varpi_{1i},\;\varpi_{2i}$ and $\varpi_{3i}$, regardless of whether estimation errors are in proximity to zero or significantly distant from it.

**Theorem 2.** By considering the error dynamic model in Eq (8) under the influence of lumped disturbance, the devised CAFNTSMFTC approach in (30) incorporating adaptive update laws in (32) guarantees the eventual convergence of the FTNTSM surface $\sigma_{i}$ and tracking errors ${\tilde{q}}_{1i}$ and ${\tilde{q}}_{2i}$ in to compact regions within a fixed-time frame.

*Proof:* The stability of the sliding phase and the reaching phase in terms of fixed-time analysis is investigated separately. To simplify the proof, we will opt for the *i*th DOF of the rigid manipulator and choose the Lyapunov function as

$$V_{2i}=\frac{1}{2}\sigma_{i}^{2}+\frac{1}{2\eta_{1i}}{\tilde{\varpi }}_{1i}^{2}+\frac{1}{2\eta_{2i}}{\tilde{\varpi }}_{2i}^{2}+\frac{1}{2\eta_{3i}}{\tilde{\varpi }}_{3i}^{2}$$ (33)

where ${\tilde{\varpi }}_{1i}=\varpi_{1i}-{\hat{\varpi }}_{1i}$, ${\tilde{\varpi }}_{2i}=\varpi_{2i}-{\hat{\varpi }}_{2i}$, and ${\tilde{\varpi }}_{3i}=\varpi_{3i}-{\hat{\varpi }}_{3i}$.

Upon calculating the time derivative of the aforementioned Eq (33) and applying Eqs 25 and (30) we obtain

$${\dot{V}}_{2i}=\sigma_{i}{\dot{\sigma }}_{i}-\frac{1}{\eta_{1i}}{\tilde{\varpi }}_{1i}{\dot{\hat{\varpi }}}_{1i}-\frac{1}{\eta_{2i}}{\tilde{\varpi }}_{2i}{\dot{\hat{\varpi }}}_{2i}-\frac{1}{\eta_{3i}}{\tilde{\varpi }}_{3i}{\dot{\hat{\varpi }}}_{3i}\;\;\;\;\;\;\;=\sigma_{i}(\left[-r_{1i}\text{si}{\text{g}}^{\gamma_{1i}}\left(\sigma_{i}\right)-r_{2i}\text{si}{\text{g}}^{\gamma_{2i}}\left(\sigma_{i}\right)\right]-r_{3i}\sigma_{i}\;-{\left(U_{adp}\right)}_{i}+D_{\iota i})\;\;\;\;\;\;\;\;\;\;\;\;\;\;\;\;\;\;\;\;-\sum_{j=1}^{3}\frac{{\tilde{\varpi }}_{ji}{\dot{\hat{\varpi }}}_{ji}}{\eta_{ji}}\;$$ (34)

$${\dot{V}}_{2i}\;\leq -\left[r_{1i}{\left|\sigma_{i}\right|}^{\gamma_{1i}+1}+r_{2i}{\left|\sigma_{i}\right|}^{\gamma_{2i}+1}\right]-r_{3i}\sigma_{i}^{2}-\frac{\sigma_{i}^{2}}{2}\left(\frac{{\hat{\varpi }}_{1i}}{\nu_{1i}^{2}}+\frac{{\hat{\varpi }}_{2i}G_{i}}{\nu_{1i}^{2}}+\frac{{\hat{\varpi }}_{3i}G_{i}^{2}}{\nu_{1i}^{2}}\right)\;\;\;\;\;\;\;\;\;\;\;\;\;+\left|\sigma_{i}\right|\left|D_{i}\right|-\sum_{j=1}^{3}\frac{1}{\eta_{ji}}{\tilde{\varpi }}_{ji}{\dot{\hat{\varpi }}}_{ji}\;$$ (35)

Using $\left|\sigma_{i}\right|\left|D_{i}\right|\;\leq \frac{{\left|\sigma_{i}\right|}^{2}{\left|D_{i}\right|}^{2}}{2\nu_{1i}^{2}}+\frac{\nu_{1i}^{2}}{2}$ and the adaptive laws in (32), we can deduce that

$${\dot{V}}_{2i}\;\leq -\left[r_{1i}{\left|\sigma_{i}\right|}^{\gamma_{1i}+1}+r_{2i}{\left|\sigma_{i}\right|}^{\gamma_{2i}+1}\right]-r_{3i}\sigma_{i}^{2}+\frac{{\left|\sigma_{i}\right|}^{2}}{2\nu_{1i}^{2}}{\left|D_{i}\right|}^{2}-\frac{\sigma_{i}^{2}}{2}\left(\frac{{\hat{\varpi }}_{1i}}{\nu_{1i}^{2}}+\frac{{\hat{\varpi }}_{2i}G_{i}}{\nu_{1i}^{2}}+\frac{{\hat{\varpi }}_{3i}G_{i}^{2}}{\nu_{1i}^{2}}\right)\;\;\;\;\;\;\;\;\;\;\;\;\;+\frac{\nu_{1i}^{2}}{2}\;-\frac{{\tilde{\varpi }}_{1i}}{\eta_{1i}}\left(-\nu_{2i}{\hat{\varpi }}_{1i}^{\gamma_{2i}}-\nu_{5i}{\hat{\varpi }}_{1i}^{\gamma_{1i}}+\frac{\eta_{1i}|\sigma_{i}{|}^{2}}{2\nu_{1i}^{2}}\right)\;-\frac{{\tilde{\varpi }}_{2i}}{\eta_{2i}}\left(-\nu_{3i}{\hat{\varpi }}_{2i}^{\gamma_{2i}}-\nu_{6i}{\hat{\varpi }}_{2i}^{\gamma_{1i}}+\frac{\eta_{2i}|\sigma_{i}{|}^{2}G_{i}}{2\nu_{1i}^{2}}\right)\;\;\;\;\;\;\;\;\;\;-\frac{{\tilde{\varpi }}_{3i}}{\eta_{3i}}\left(-\nu_{4i}{\hat{\varpi }}_{3i}^{\gamma_{2i}}-\nu_{7i}{\hat{\varpi }}_{3i}^{\gamma_{1i}}+\frac{\eta_{3i}|\sigma_{i}{|}^{2}G_{i}^{2}}{2\nu_{1i}^{2}}\right)\;\;\;\leq -\left[r_{1i}{\left|\sigma_{i}\right|}^{\gamma_{1i}+1}+r_{2i}{\left|\sigma_{i}\right|}^{\gamma_{2i}+1}\right]-r_{3i}\sigma_{i}^{2}+\frac{{\left|\sigma_{i}\right|}^{2}}{2}\left(\frac{\varpi_{1i}}{\nu_{1i}^{2}}+\frac{\varpi_{2i}G_{i}}{\nu_{1i}^{2}}+\frac{\varpi_{3i}G_{i}^{2}}{\nu_{1i}^{2}}\right)\;\;\;\;\;\;\;\;\;-\frac{{\left|\sigma_{i}\right|}^{2}}{2}\left(\frac{{\hat{\varpi }}_{1i}}{\nu_{1i}^{2}}+\frac{{\hat{\varpi }}_{2i}G_{i}}{\nu_{1i}^{2}}+\frac{{\hat{\varpi }}_{3i}G_{i}^{2}}{\nu_{1i}^{2}}\right)+\frac{\nu_{1i}^{2}}{2}\;-\frac{{\tilde{\varpi }}_{1i}}{\eta_{1i}}\left(-\nu_{2i}{\hat{\varpi }}_{1i}^{\gamma_{2i}}-\nu_{5i}{\hat{\varpi }}_{1i}^{\gamma_{1i}}+\frac{\eta_{1i}|\sigma_{i}{|}^{2}}{2\nu_{1i}^{2}}\right)\;\;\;\;\;\;\;\;\;-\frac{{\tilde{\varpi }}_{2i}}{\eta_{2i}}\left(-\nu_{3i}{\hat{\varpi }}_{2i}^{\gamma_{2i}}-\nu_{6i}{\hat{\varpi }}_{2i}^{\gamma_{1i}}+\frac{\eta_{2i}|\sigma_{i}{|}^{2}G_{i}}{2\nu_{1i}^{2}}\right)-\frac{{\tilde{\varpi }}_{3i}}{\eta_{3i}}\left(-\nu_{4i}{\hat{\varpi }}_{3i}^{\gamma_{2i}}-\nu_{7i}{\hat{\varpi }}_{3i}^{\gamma_{1i}}+\frac{\eta_{3i}|\sigma_{i}{|}^{2}G_{i}^{2}}{2\nu_{1i}^{2}}\right)\;\leq -\left[r_{1i}{\left|\sigma_{i}\right|}^{\gamma_{1i}+1}+r_{2i}{\left|\sigma_{i}\right|}^{\gamma_{2i}+1}\right]+\frac{{\left|\sigma_{i}\right|}^{2}}{2}\left(\frac{\varpi_{1i}}{\nu_{1i}^{2}}+\frac{\varpi_{2i}G_{i}}{\nu_{1i}^{2}}+\frac{\varpi_{3i}G_{i}^{2}}{\nu_{1i}^{2}}\right)\;\;\;\;\;\;-\frac{{\left|\sigma_{i}\right|}^{2}}{2}\left(\frac{{\hat{\varpi }}_{1i}}{\nu_{1i}^{2}}+\frac{{\hat{\varpi }}_{2i}G_{i}}{\nu_{1i}^{2}}+\frac{{\hat{\varpi }}_{3i}G_{i}^{2}}{\nu_{1i}^{2}}\right)+\frac{\nu_{1i}^{2}}{2}\;\;\;-\frac{{\tilde{\varpi }}_{1i}|\sigma_{i}{|}^{2}}{2\nu_{1i}^{2}}+\frac{\nu_{2i}}{\eta_{1i}}{\tilde{\varpi }}_{1i}{\hat{\varpi }}_{1i}^{\gamma_{2i}}+\frac{\nu_{5i}}{\eta_{1i}}{\tilde{\varpi }}_{1i}{\hat{\varpi }}_{1i}^{\gamma_{1i}}\;\;\;\;\;\;-\frac{{\tilde{\varpi }}_{2i}|\sigma_{i}{|}^{2}G_{i}}{2\nu_{1i}^{2}}+\frac{\nu_{3i}}{\eta_{2i}}{\tilde{\varpi }}_{2i}{\hat{\varpi }}_{2i}^{\gamma_{2i}}+\frac{\nu_{6i}}{\eta_{2i}}{\tilde{\varpi }}_{2i}{\hat{\varpi }}_{2i}^{\gamma_{1i}}-\frac{{\tilde{\varpi }}_{3i}|\sigma_{i}{|}^{2}G_{i}^{2}}{2\nu_{1i}^{2}}+\frac{\nu_{4i}}{\eta_{3i}}{\tilde{\varpi }}_{3i}{\hat{\varpi }}_{3i}^{\gamma_{2i}}+\frac{\nu_{7i}}{\eta_{3i}}{\tilde{\varpi }}_{3i}{\hat{\varpi }}_{3i}^{\gamma_{1i}}\;=-\left[r_{1i}{\left|\sigma_{i}\right|}^{\gamma_{1i}+1}+r_{2i}{\left|\sigma_{i}\right|}^{\gamma_{2i}+1}\right]+\frac{\nu_{2i}}{\eta_{1i}}{\tilde{\varpi }}_{1i}{\hat{\varpi }}_{1i}^{\gamma_{2i}}+\frac{\nu_{3i}}{\eta_{2i}}{\tilde{\varpi }}_{2i}{\hat{\varpi }}_{2i}^{\gamma_{2i}}+\frac{\nu_{4i}}{\eta_{3i}}{\tilde{\varpi }}_{3i}{\hat{\varpi }}_{3i}^{\gamma_{2i}}+\frac{\nu_{5i}}{\eta_{1i}}{\tilde{\varpi }}_{1i}{\hat{\varpi }}_{1i}^{\gamma_{1i}}\;\;\;\;\;\;+\frac{\nu_{6i}}{\eta_{2i}}{\tilde{\varpi }}_{2i}{\hat{\varpi }}_{2i}^{\gamma_{1i}}\;+\frac{\nu_{7i}}{\eta_{3i}}{\tilde{\varpi }}_{3i}{\hat{\varpi }}_{3i}^{\gamma_{1i}}+\frac{\nu_{1i}^{2}}{2}\;\;$$ (36)

It is obtained from Lemma 4 and 5 that

$$\frac{\nu_{\left(j+4\right)i}{\tilde{\varpi }}_{ji}{\hat{\varpi }}_{ji}^{\gamma_{1i}}}{n_{ji}}\leq \frac{\nu_{\left(j+4\right)i}\left(\varpi_{ji}^{\gamma_{1i}+1}-{\hat{\varpi }}_{ji}^{\gamma_{1i}+1}\right)}{n_{ji}\left(\gamma_{1i}+1\right)}\;=\frac{\nu_{\left(j+4\right)i}\left(\varpi_{ji}^{\gamma_{1i}+1}-{\left(\varpi_{ji}-{\tilde{\varpi }}_{ji}\right)}^{\gamma_{1i}+1}\right)}{n_{ji}\left(\gamma_{1i}+1\right)}\;\leq \frac{\nu_{\left(j+4\right)i}\left(2\varpi_{ji}^{\gamma_{1i}+1}-{\tilde{\varpi }}_{ji}^{\gamma_{1i}+1}\right)}{n_{ji}\left(\gamma_{1i}+1\right)},\;(j=1,2,3)$$ (37)

and similarly we have

$$\frac{\nu_{\left(j+1\right)i}{\tilde{\varpi }}_{ji}{\hat{\varpi }}_{ji}^{\gamma_{2i}}}{n_{ji}}\leq \frac{\nu_{\left(j+1\right)i}\left(\varpi_{ji}^{\gamma_{2i}+1}-{\hat{\varpi }}_{ji}^{\gamma_{2i}+1}\right)}{n_{ji}\left(\gamma_{2i}+1\right)}\;\;\;\;\;\;\;\;\;\;\;\;\;\;\;\;\;\;\;\;\;\;\;\;\;\;\;\;\;\;\;\;\;\;=\frac{\nu_{\left(j+1\right)i}\left(\varpi_{ji}^{\gamma_{2i}+1}-{\left(\varpi_{ji}-{\tilde{\varpi }}_{ji}\right)}^{\gamma_{2i}+1}\right)}{n_{ji}\left(\gamma_{2i}+1\right)}\;\;\;\;\;\;\;\;\;\;\;\;\;\;\;\;\;\;\;\;\;\;\;\;\;\;\;\;\;\;\;\;\;\;\leq \frac{\nu_{\left(j+1\right)i}\left(2\varpi_{ji}^{\gamma_{2i}+1}-{\tilde{\varpi }}_{ji}^{\gamma_{2i}+1}\right)}{n_{ji}\left(\gamma_{2i}+1\right)}\;$$ (38)

where j=1,2,3. Substituting (37) and (38) we can write (36) as

$${\dot{V}}_{2i}\leq -\left[r_{1i}{\left|\sigma_{i}\right|}^{\gamma_{1i}+1}+r_{2i}{\left|\sigma_{i}\right|}^{\gamma_{2i}+1}\right]+\frac{2\nu_{2i}\varpi_{1i}^{\gamma_{2i}+1}}{n_{1i}\left(\gamma_{2i}+1\right)}-\frac{\nu_{2i}{\tilde{\varpi }}_{1i}^{\gamma_{2i}+1}}{n_{1i}\left(\gamma_{2i}+1\right)}\;\;\;\;\;\;\;\;\;+\frac{2\nu_{3i}\varpi_{2i}^{\gamma_{2i}+1}}{n_{2i}\left(\gamma_{2i}+1\right)}-\frac{\nu_{3i}{\tilde{\varpi }}_{2i}^{\gamma_{2i}+1}}{n_{2i}\left(\gamma_{2i}+1\right)}+\frac{2\nu_{4i}\varpi_{3i}^{\gamma_{2i}+1}}{n_{3i}\left(\gamma_{2i}+1\right)}-\frac{\nu_{4i}{\tilde{\varpi }}_{3i}^{\gamma_{2i}+1}}{n_{3i}\left(\gamma_{2i}+1\right)}\;\;\;\;\;\;\;+\frac{\nu_{1i}^{2}}{2}+\frac{2\nu_{5i}\varpi_{1i}^{\gamma_{1i}+1}}{n_{1i}\left(\gamma_{1i}+1\right)}-\frac{\nu_{5i}{\tilde{\varpi }}_{1i}^{\gamma_{1i}+1}}{n_{1i}\left(\gamma_{1i}+1\right)}+\frac{2\nu_{6i}\varpi_{2i}^{\gamma_{1i}+1}}{n_{2i}\left(\gamma_{1i}+1\right)}-\frac{\nu_{6i}{\tilde{\varpi }}_{2i}^{\gamma_{1i}+1}}{n_{2i}\left(\gamma_{1i}+1\right)}\;\;\;\;\;\;\;+\frac{2\nu_{7i}\varpi_{3i}^{\gamma_{1i}+1}}{n_{3i}\left(\gamma_{1i}+1\right)}-\frac{\nu_{7i}{\tilde{\varpi }}_{3i}^{\gamma_{1i}+1}}{n_{3i}\left(\gamma_{1i}+1\right)}\;\;\;\;=-(r_{1i}{\left|\sigma_{i}\right|}^{\gamma_{1i}+1}+\frac{\nu_{5i}{\tilde{\varpi }}_{1i}^{\gamma_{1i}+1}}{n_{1i}\left(\gamma_{1i}+1\right)}+\frac{\nu_{6i}{\tilde{\varpi }}_{2i}^{\gamma_{1i}+1}}{n_{2i}\left(\gamma_{1i}+1\right)}+\frac{\nu_{7i}{\tilde{\varpi }}_{3i}^{\gamma_{1i}+1}}{n_{3i}\left(\gamma_{1i}+1\right)})\;\;\;\;\;\;\;\;-\left(r_{2i}{\left|\sigma_{i}\right|}^{\gamma_{2i}+1}+\frac{\nu_{2i}{\tilde{\varpi }}_{1i}^{\gamma_{2i}+1}}{n_{1i}\left(\gamma_{2i}+1\right)}+\frac{\nu_{3i}{\tilde{\varpi }}_{2i}^{\gamma_{2i}+1}}{n_{2i}\left(\gamma_{2i}+1\right)}+\frac{\nu_{4i}{\tilde{\varpi }}_{3i}^{\gamma_{2i}+1}}{n_{3i}\left(\gamma_{2i}+1\right)}\right)\;\;\;\;\;\;\;\;\;+\frac{2\nu_{5i}\varpi_{1i}^{\gamma_{1i}+1}}{n_{1i}\left(\gamma_{1i}+1\right)}\;+\frac{2\nu_{2i}\varpi_{1i}^{\gamma_{2i}+1}}{n_{1i}\left(\gamma_{2i}+1\right)}+\frac{2\nu_{6i}\varpi_{2i}^{\gamma_{1i}+1}}{n_{2i}\left(\gamma_{1i}+1\right)}+\frac{2\nu_{3i}\varpi_{2i}^{\gamma_{2i}+1}}{n_{2i}\left(\gamma_{2i}+1\right)}\;\;\;\;\;\;\;\;\;\;\;+\frac{2\nu_{7i}\varpi_{3i}^{\gamma_{1i}+1}}{n_{3i}\left(\gamma_{1i}+1\right)}+\frac{2\nu_{4i}\varpi_{3i}^{\gamma_{2i}+1}}{n_{3i}\left(\gamma_{2i}+1\right)}+\frac{\nu_{1i}^{2}}{2}\;\;\;\;\;\;\leq -\Upsilon_{1i}V_{2i}^{\frac{\gamma_{1i}+1}{2}}-\Upsilon_{2i}V_{2i}^{\frac{\gamma_{2i}+1}{2}}+\Upsilon_{3i}\;-\Upsilon_{4i}\sum_{j=1}^{2}r_{ji}{\left|\sigma_{i}\right|}^{\gamma_{ji}+1}\;$$ (39)

where $\Upsilon_{1i}=\sqrt{2^{\gamma_{1i}+1}}\min(r_{1i}\left(1-\Upsilon_{4i}\right),\frac{\nu_{5i}\sqrt{n_{1i}^{\gamma_{1i}-1}}}{\left(\gamma_{1i}+1\right)},\frac{\nu_{6i}\sqrt{n_{2i}^{\gamma_{1i}-1}}}{\left(\gamma_{1i}+1\right)},\frac{\nu_{7i}\sqrt{n_{3i}^{\gamma_{1i}-1}}}{\left(\gamma_{1i}+1\right)})$, $\Upsilon_{2i}=2^{1-\gamma_{2i}}\sqrt{2^{\gamma_{2i}+1}}$ $\min(r_{2i}\left(1-\Upsilon_{4i}\right),\frac{\nu_{2i}n_{1i}^{\frac{\gamma_{2i}-1}{2}}}{\left(\gamma_{2i}+1\right)},\frac{\nu_{3i}n_{2i}^{\frac{\gamma_{2i}-1}{2}}}{\left(\gamma_{2i}+1\right)},\frac{\nu_{4i}n_{3i}^{\frac{\gamma_{2i}-1}{2}}}{\left(\gamma_{2i}+1\right)})$ and $\Upsilon_{3i}=\frac{2\nu_{5i}\varpi_{1i}^{\gamma_{1i}+1}}{n_{1i}\left(\gamma_{1i}+1\right)}\mathrm{\backslash break}$ $+\;\frac{2\nu_{2i}\varpi_{1i}^{\gamma_{2i}+1}}{n_{1i}\left(\gamma_{2i}+1\right)}+\frac{2\nu_{6i}\varpi_{2i}^{\gamma_{1i}+1}}{n_{2i}\left(\gamma_{1i}+1\right)}+\frac{2\nu_{3i}\varpi_{2i}^{\gamma_{2i}+1}}{n_{2i}\left(\gamma_{2i}+1\right)}+\frac{2\nu_{7i}\varpi_{3i}^{\gamma_{1i}+1}}{n_{3i}\left(\gamma_{1i}+1\right)}+\frac{2\nu_{4i}\varpi_{3i}^{\gamma_{2i}+1}}{n_{3i}\left(\gamma_{2i}+1\right)}+\frac{\nu_{1i}^{2}}{2}.$

If $\left|\sigma_{i}\right|$ > $\Omega_{\sigma_{i}}=\min\left(\sqrt[{\gamma_{1i}+1}]{\frac{\Upsilon_{3i}}{r_{1i}\Upsilon_{4i}}},\;\sqrt[{\gamma_{2i}+1}]{\frac{\Upsilon_{3i}}{r_{2i}\Upsilon_{4i}}}\right),$ Then the above inequality (39) can be restated as given below

$${\dot{V}}_{2i}\;\leq -\Upsilon_{1i}V_{2i}^{\frac{\gamma_{1i}+1}{2}}-\Upsilon_{2i}V_{2i}^{\frac{\gamma_{2i}+1}{2}}$$ (40)

From lemma 1 and the inequality (40), it can be deduced that the sliding variable can be brought within the $\Omega_{1}=\left\{\sigma_{i}\left|\sigma_{i}\right|\leq \Omega_{\sigma_{i}}=\min\left(\sqrt[{\gamma_{1i}+1}]{\frac{\Upsilon_{3i}}{r_{1i}\Upsilon_{4i}}},\;\sqrt[{\gamma_{2i}+1}]{\frac{\Upsilon_{3i}}{r_{2i}\Upsilon_{4i}}}\right)\right\},$ range within a fixed-time frame. Additionally, the time it takes to achieve this state is stated as

$$T_{s}\;\leq \frac{2}{\Upsilon_{1i}\left(1-\gamma_{1i}\right)}+\frac{2}{\Upsilon_{2i}\left(\gamma_{2i}-1\right)}$$ (41)

The preceding analysis primarily pertains to the reaching phase. Moving forward, our next focus will be to explore the extent of error convergence during the sliding phase. Based on the analysis conducted during the reaching phase, it has been established that the sliding variable $\sigma_{i}$ is compelled to reach $\Omega_{1}$ within a fixed-time frame. To solidify the proof, we need to examine the following three cases, taking into account the definition of $\sigma_{i}$ in Eq (12). When the ${\bar{\sigma }}_{i}=0$ condition is met, it is evident from the prior analysis that

$$\sigma_{i}={\tilde{q}}_{2i}+\alpha_{1i}\text{si}{\text{g}}^{\frac{\Theta_{1}}{\Theta_{2}}}\left({\tilde{q}}_{1i}\right)+\alpha_{2i}\text{si}{\text{g}}^{\frac{\Theta_{3}}{\Theta_{4}}}+\alpha_{3i}{\tilde{q}}_{1i}=0$$ (42)

It is easily deduced from Theorem 1 that achieving ${\tilde{q}}_{1i}=0$ and ${\tilde{q}}_{2i}=0$ occurs within a fixed time frame. When ${\bar{\sigma }}_{i}\neq 0$ and $\left|{\tilde{q}}_{1i}\right|\geq \Delta_{i}$ we have

$${\tilde{q}}_{2i}+\alpha_{1i}\text{si}{\text{g}}^{\frac{\Theta_{1}}{\Theta_{2}}}\left({\tilde{q}}_{1i}\right)+\alpha_{2i}\text{si}{\text{g}}^{\frac{\Theta_{3}}{\Theta_{4}}}\left({\tilde{q}}_{1i}\right)+\alpha_{3i}{\tilde{q}}_{1i}=\gamma_{\sigma i},\;$$ (43)

$\;\left|\;\gamma_{\sigma i}\right|\leq \Omega_{\sigma_{i}}$. Eq (43) can be expressed in an alternative manner and can be reformulated as

$${\tilde{q}}_{2i}+\left(\alpha_{1i}-\frac{\gamma_{\sigma i}}{\text{si}{\text{g}}^{\frac{\Theta_{1}}{\Theta_{2}}}\left({\tilde{q}}_{1i}\right)}\right)\text{si}{\text{g}}^{\frac{\Theta_{1}}{\Theta_{2}}}\left({\tilde{q}}_{1i}\right)+\alpha_{2i}\text{si}{\text{g}}^{\frac{\Theta_{3}}{\Theta_{4}}}\left({\tilde{q}}_{1i}\right)+\alpha_{3i}{\tilde{q}}_{1i}=0,$$ (44)

$${\tilde{q}}_{2i}+\alpha_{1i}\text{si}{\text{g}}^{\frac{\Theta_{1}}{\Theta_{2}}}\left({\tilde{q}}_{1i}\right)+\alpha_{3i}{\tilde{q}}_{1i}+\left(\alpha_{2i}-\frac{\gamma_{\sigma i}}{\text{si}{\text{g}}^{\frac{\Theta_{3}}{\Theta_{4}}}\left({\tilde{q}}_{1i}\right)}\right)\text{si}{\text{g}}^{\frac{\Theta_{3}}{\Theta_{4}}}\left({\tilde{q}}_{1i}\right)=0,$$ (45)

$${\tilde{q}}_{2i}+\alpha_{1i}\text{si}{\text{g}}^{\frac{\Theta_{1}}{\Theta_{2}}}\left({\tilde{q}}_{1i}\right)+\alpha_{2i}\text{si}{\text{g}}^{\frac{\Theta_{3}}{\Theta_{4}}}\left({\tilde{q}}_{1i}\right)+\left(\alpha_{3i}-\frac{\gamma_{\sigma i}}{\left|{\tilde{q}}_{1i}\right|sign\left({\tilde{q}}_{1i}\right)}\right){\tilde{q}}_{1i}=0.$$ (46)

As per the expression given in Eq (46), when the $\alpha_{3i}$ − $\frac{\gamma_{\sigma i}}{\left|{\tilde{q}}_{1i}\right|sign\left({\tilde{q}}_{1i}\right)}$ > 0 holds true, Eq (46) preserves the structure of the FTNTSM surface (12), and the system state can achieve convergence at a fixed-time to

$$\left|{\tilde{q}}_{1i}\right|\leq \frac{\Omega_{\sigma_{i}}}{\alpha_{3i}}$$ (47)

By performing an analogous analysis for Eqs 44 and (45), the system variables will be guided towards

$$\left|{\tilde{q}}_{1i}\right|\leq {\left(\frac{\Omega_{\sigma_{i}}}{\alpha_{1i}}\right)}^{\frac{\Theta_{2}}{\Theta_{1}}}$$ (48)

$$\left|{\tilde{q}}_{1i}\right|\leq {\left(\frac{\Omega_{\sigma_{i}}}{\alpha_{2i}}\right)}^{\frac{\Theta_{4}}{\Theta_{3}}}$$ (49)

in fixed-time. Moreover, based on the analysis provided above, the variable ${\tilde{q}}_{1i}$ will attain fixed-time convergence to

$$\left|{\tilde{q}}_{1i}\right|\leq \Omega_{i}=\max\left(\Delta_{i},\;\Pi_{i}\right).$$ (50)

where $\Pi_{i}=\min\{\left(\frac{\Omega_{\sigma_{i}}}{\alpha_{3i}}\right),\;\;{\left(\frac{\Omega_{\sigma_{i}}}{\alpha_{2i}}\right)}^{\frac{\Theta_{4}}{\Theta_{3}}},\;\;{\left(\frac{\Omega_{\sigma_{i}}}{\alpha_{1i}}\right)}^{\frac{\Theta_{2}}{\Theta_{1}}}\}$. If ${\bar{\sigma }}_{i}\neq 0,\;\left|{\tilde{q}}_{1i}\right|$ < $\Delta_{i}$, the error convergence region is already encompassed within the above term (50) at this time. To sum up, the error can reach convergence in a fixed-time frame to the vicinity of the equilibrium point.

**Remark 2.** In contrast to the fixed-time control approaches outlined in the studies by [25,28], the control law presented in Eq (30) does not necessitate prior knowledge of uncertainties thanks to the incorporation of adaptive techniques. It is important to highlight that even though the adaptive fixed-time control strategies in [35,39] introduce a boundary layer concept, their robustness to uncertainties diminishes, and control performance deteriorates within this boundary layer. In comparison to the aforementioned literature, our proposed control method estimates the square of the upper bound of uncertainty, eliminating the need for a boundary layer. Consequently, the proposed control approach enhances robustness and offers improved control accuracy.

**Remark 3.** It is important to emphasize that the control parameters $\Theta_{i}(i=1,\;2,3,4),$ $\;\;\alpha_{j},\;r_{j}\;(j=1,\;2,3),\;\;$ and $\gamma_{l}\;\left(l=1,\;2\right)$ significantly affect both the accuracy of the pointing and the required control effort. To achieve desirable performance, these gains should be carefully tuned according to the following guidelines.

(a) The settling time expressions reveal that the parameters $\Theta_{i}(i=1,\;2,3,4),$ $\;\;\gamma_{l}\;\left(l=1,\;2\right)\;$ play a crucial role in determining the system’s convergence rate and accuracy.
(b) Parameters *j*_1*i*_ and *j*_2*i*_ guarantee the continuity and differentiability of the function $\sigma_{d}{\left({\tilde{q}}_{1i}\right)}_{i}$. When ${\bar{\sigma }}_{i}\neq 0$ and $\left|{\tilde{q}}_{1i}\right|\;<\;\Delta_{i},$ the error states seamlessly shift from terminal sliding mode to a general sliding manifold, thereby avoiding singularity issue in the case of ${\bar{\sigma }}_{i}\neq 0$ and ${\tilde{q}}_{1i}=0$. Furthermore, by selecting an appropriately small $\Delta_{i}$ such that $\left|{\tilde{q}}_{1i}\right|\geq \Delta_{i},$ as the sliding mode variable approaches zero, the error ${\tilde{q}}_{1i}$ will converge along $\bar{\sigma }$. As a result, the convergence of ${\tilde{q}}_{1i}$ within a fixed-time can be ensured.
(c) Higher values of the parameters $\alpha_{j},\;r_{j}\;(j=1,\;2,3)\;\;$can lead to faster convergence, but it can also cause increased overshoot and higher control effort.

**Fig 3:**
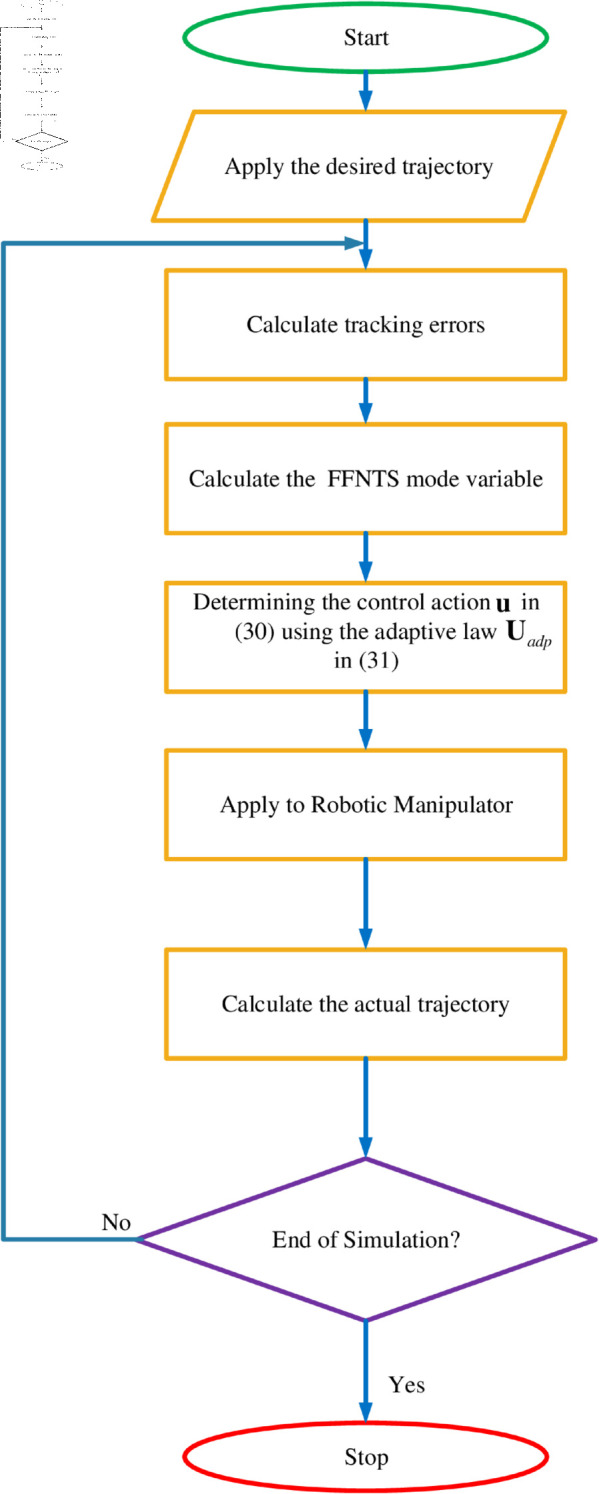
Workflow of the proposed control system.

In order to achieve the required convergence rate and control accuracy, it is crucial to choose the correct control gains. Unfortunately, there is no established method for selecting these gains, and they are often chosen based on trial and error until adequate performance is reached. As a result, identifying a tuning mechanism to adjust the control gains for optimal performance is a significant challenge and a goal for future research. The control process is depicted in the flowchart in Fig 3.

## 4 Findings from simulations and subsequent discussions

To assess the proposed control method’s efficiency, we employ a PUMA560 robot, a widely recognized robot commonly utilized as a standard in research and development. Although the PUMA560 robot boasts a total of six joints, our investigation will exclusively focus on the initial three joints to maintain succinctness in the simulation results. The dynamic model of the PUMA560 conforms to the structure outlined in Eq (1), and it incorporates the nominal parameters sourced from reference [43].

The modeling of friction and the disturbance term is represented as follows:


$$\text{F}({\dot{q}}_{l})=\left[\begin{array}{c} 0.5{\dot{q}}_{l1}+\sin(3q_{l1})(\text{N.m})\\ 1.3{\dot{q}}_{l2}-1.8\sin(2q_{l2})(\text{N.m})\\ -1.8{\dot{q}}_{l3}-2\sin(q_{l3})(\text{N.m}) \end{array}\right]$$ (51)

$$1_{d}=\left[\begin{array}{c} 5.4\cos(t)+1.5\sin(t)\cos(t)(\text{N.m})\\ 1.5\cos(t)+3\cos(\text{t})(\text{N.m})\\ 2.5\sin(t)-2.2\sin(t)\cos(t)(\text{N.m}) \end{array}\right]$$ (52)

The desired trajectories of the system are selected as follows:


$$q_{d}=\left[\begin{array}{c} 0.5+\sin\left(\frac{t}{5\pi }+\frac{\pi }{2}\right)-1(\text{rad})\\ 0.5+\cos\left(\frac{t}{5\pi }\right)-1(\text{rad)}\\ -0.5+\sin\left(\frac{t}{5\pi }+\frac{\pi }{2}\right)(\text{rad}) \end{array}\right]$$ (53)

The simulations have been carried out by employing the MATLAB/Simulink software, making use of the Runge-Kutta solver, with a selected time step of 1 millisecond (ms). Our simulation experiments have considered three distinct scenarios. In the first and second scenarios, we evaluate the effectiveness of the suggested controller under normal and fault conditions, respectively. In the third scenario, we assess the performance of the proposed controller by subjecting it to testing with various initial state values. Furthermore, we have conducted a comparison between the CAFNTSMFTC we propose and other state-of-the-art controllers, highlighting the superior performance of the CAFNTSMFTC controller in both normal and faulty operational scenarios. Here are the defined parameters for the proposed CAFNTSMFTC controller: $\Theta_{1}=7,\;\Theta_{2}=5,$ $\;\Theta_{3}=3,\;\Theta_{4}=5,\;\alpha_{1i}=0.4,$ $\alpha_{2i}=0.4,\alpha_{3i}\;=\;0.5,$ $\Delta_{i}=0.01,$ *r*_1*i*_ = 30, $\nu_{6i}=0.02,$ *r*_2*i*_ = 30, *r*_3*i*_ = 50, $\gamma_{1i}=0.4$, $\gamma_{2i}=1.4$, $\nu_{1i}=3$, $\nu_{2i}=0.05$, $\nu_{3i}=0.07$, $\nu_{4i}=0.01$, $\nu_{5i}=0.04$, $\nu_{7i}=0.06$, $\eta_{1i}=0.3$, $\eta_{2i}=0.35$, $\eta_{3i}=0.3$, ${\hat{\varpi }}_{1i}\left(0\right)=0.01$, ${\hat{\varpi }}_{2i}\left(0\right)=0.01$, ${\hat{\varpi }}_{3i}\left(0\right)=0.01.$ The parameters utilized in this simulation are selected either through trial-and-error process or guided by prior experience. The goal is to strike a balance between achieving rapid convergence and minimizing oscillations.

In this part of the simulation, we presume that the system is functioning under typical operational conditions, accounting for expected uncertainties and disturbances. To showcase the enhanced effectiveness of the suggested CAFNTSMFTC controller, we conduct a comparative evaluation against contemporary controllers that have been introduced to enhance the trajectory tracking performance of robot manipulators. These controllers include computed torque control (CTC) [12], and adaptive nonsingular fast terminal sliding-mode control (ANFTSMC) [44]. The design of the CTC, is represented in [12] with parameters *K*_*p*_ = 150 and *K*_*d*_ = 20. Based on the research conducted by [44], ANFTSMC has been formulated as

$$\sigma ={\tilde{q}}_{1}+\alpha_{1}{\left|{\tilde{q}}_{1}\right|}^{\alpha }\text{sign}\left({\tilde{q}}_{1}\right)+\alpha_{2}{\left|{\tilde{q}}_{2}\right|}^{\beta }\text{sign}\left({\tilde{q}}_{2}\right)$$ (54)

$$u=-C{\left(\tilde{q}\right)}^{-1}(H_{\iota }(\tilde{q})+\frac{1}{\alpha_{2}\beta }{\left|{\tilde{q}}_{2}\right|}^{2-\beta }\left(1+\alpha_{1}\alpha {\left|{\tilde{q}}_{1}\right|}^{\alpha -1}\right)\text{sign}\left({\tilde{q}}_{2}\right)\;\;\;\;\;\;\;\;\;\;\;\;\;+k.\sigma +\left({\hat{b}}_{0}+{\hat{b}}_{1}\left|q_{\iota }\right|+{\hat{b}}_{2}{\left|{\dot{q}}_{\iota }\right|}^{2}+\xi \right)\mathrm{sign}(\sigma ))$$ (55)

$${\dot{\hat{b}}}_{0}=\lambda_{0}\left|\sigma \right|{\left|{\tilde{q}}_{2}\right|}^{\beta -1}\;{\dot{\hat{b}}}_{1}=\lambda_{1}\left|\sigma \right|\left|q_{\iota }\right|{\left|{\tilde{q}}_{2}\right|}^{\beta -1}\;{\dot{\hat{b}}}_{2}=\lambda_{2}\left|\sigma \right|{\left|{\dot{q}}_{\iota }\right|}^{2}{\left|{\tilde{q}}_{2}\right|}^{\beta -1}\;$$ (56)

**Fig 4:**
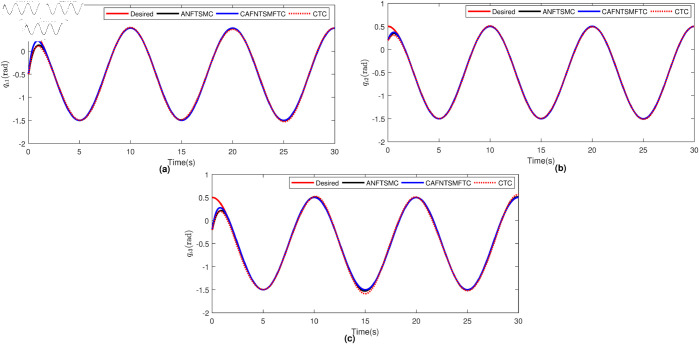
Position tracking performance under faultless operation.

**Fig 5:**
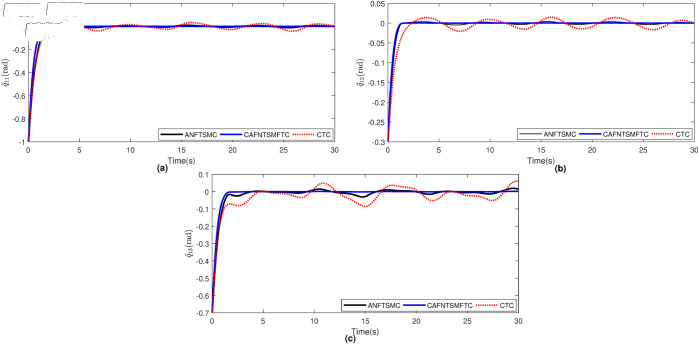
Trajectory tracking errors under faultless operation.

The parameters of ANFTSMC are selected as follows: $\alpha =2,\;\beta =5/3\;,\;\xi =0.5,\;\alpha_{1}=\alpha_{2}=1,\;k=100,\;\lambda_{0}=\lambda_{1}=\lambda_{2}\;=\;0.01.$ The initial conditions for joint positions have been selected as $q_{\iota 1}\left(0\right)=-0.5\text{rad}$,$q_{\iota 2}\left(0\right)=0.2\text{rad}$, and $q_{\iota 3}\left(0\right)=-0.2\text{rad}$. To facilitate a more straightforward comparison, we present the position tracking performance and the position tracking error for each joint of the manipulator robot in Figs 4 and 5, respectively. By examining Figs 4 and 5, it becomes evident that the CTC exhibits subpar tracking performance for the system. This deficiency can be attributed to the challenges posed by uncertainties and disturbances, which the CTC does not manage effectively within this particular system. Because of the robust characteristics of SMC in handling disturbances and uncertainties, the ANFTSMC demonstrates superior performance compared to the CTC, as evident in Figs 4 and 5. Furthermore, the proposed CAFNTSMFTC outperforms both the CTC and ANFTSMC controllers in terms of position tracking and trajectory tracking error performance, as shown in Figs 4 and 5. Subsequently, we evaluate the fault-tolerant capabilities of the proposed controller. Initially, it was compared with two other controllers under fault-free operation. In addition to these, the Li fixed-time controller [45] is now also utilized for comparison under conditions where actuators are affected by faults. To simulate the repercussions of faults within the system, we posit the presence of the following fault function:

$$\Omega_{2}({𝐪}_{\iota },{\dot{q}}_{\iota },\tau )=\left[25\sin\left(q_{\iota 1}q_{\iota 2}\right)+1.5\cos\left({\dot{q}}_{\iota 1}q_{\iota 2}\right)+2.5\cos\left({\dot{q}}_{\iota 1}{\dot{q}}_{\iota 2}\right)\;\;\;\;\;\;\;\;\;\;\;\;\;\;\;\;\;\;\;\;\;\;\;\;\;\;\;\;\;\;\;\;\;\;\;0.3\sin\left(t\right)u_{2},\;15\sin\left(q_{\iota 3}q_{\iota 1}\right)+1.2\cos\left({\dot{q}}_{\iota 2}q_{\iota 2}\right)+2.5\cos\left({\dot{q}}_{\iota 2}{\dot{q}}_{\iota 3}\right)\;\right]\;t\geq 20s$$ (57)

**Fig 6:**
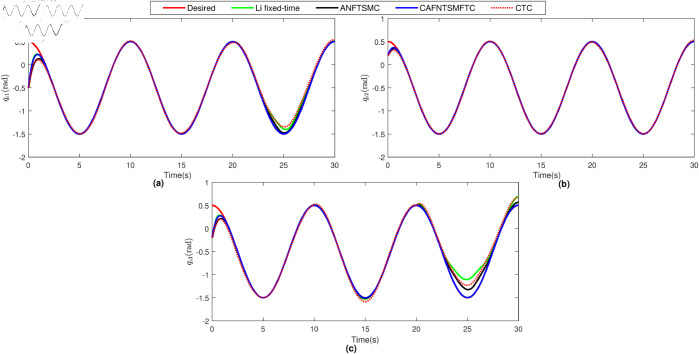
Position tracking performance under fault operation.

**Fig 7:**
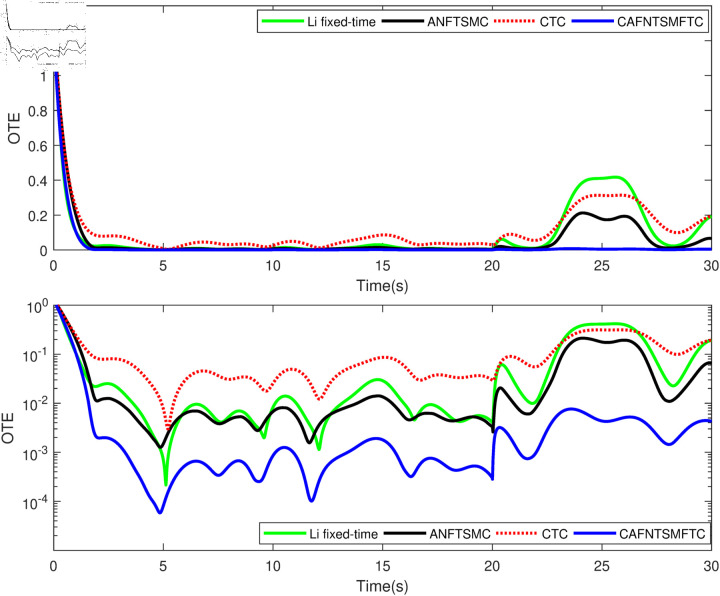
Overall tracking errors under fault operation.

**Fig 8:**
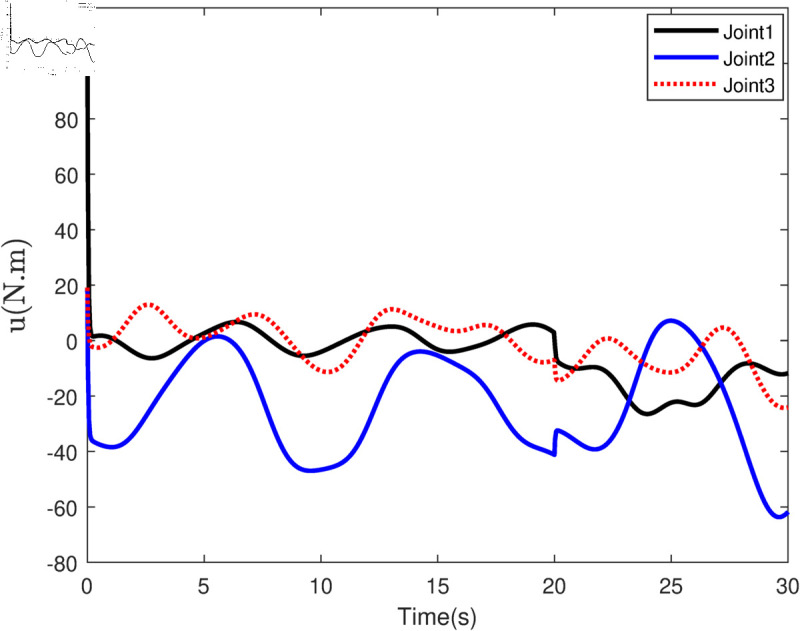
Control input of the CTC Controller.

**Fig 9:**
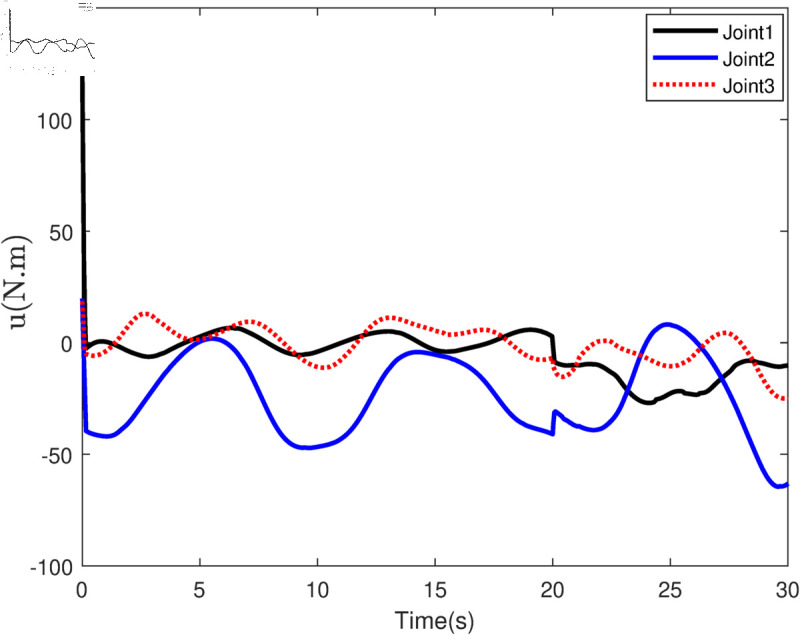
Control input of the ANFTSMC Controller.

**Fig 10:**
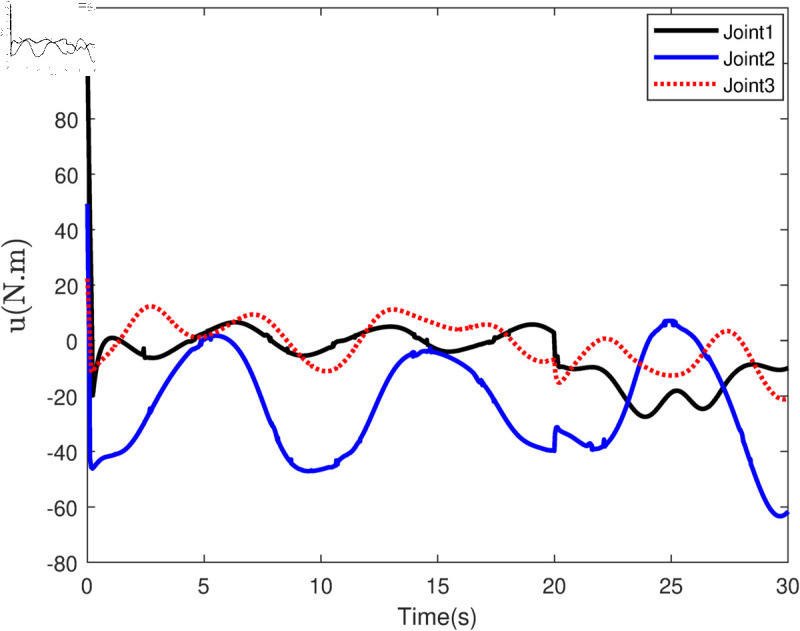
Control input of the Li fixed-time Controller.

**Fig 11:**
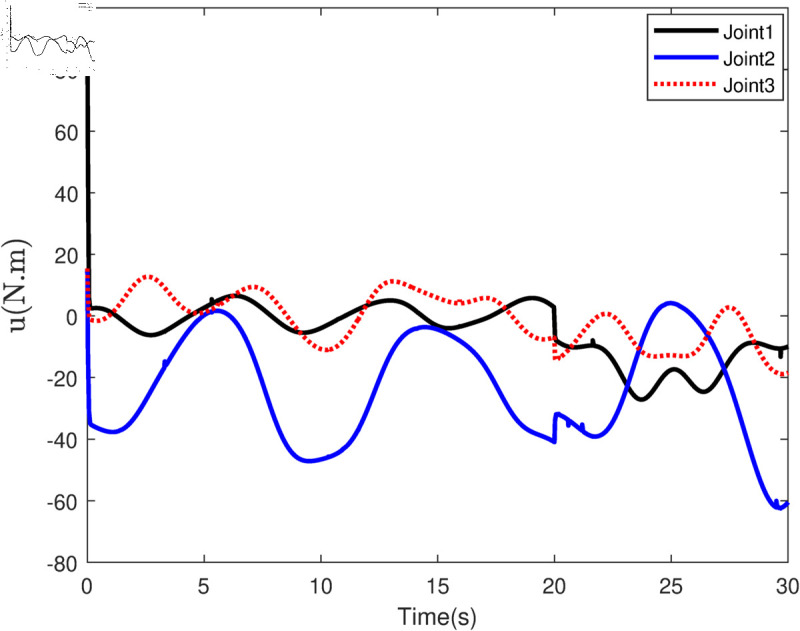
Control input of the CAFNTSMFTC Controller.

**Fig 12:**
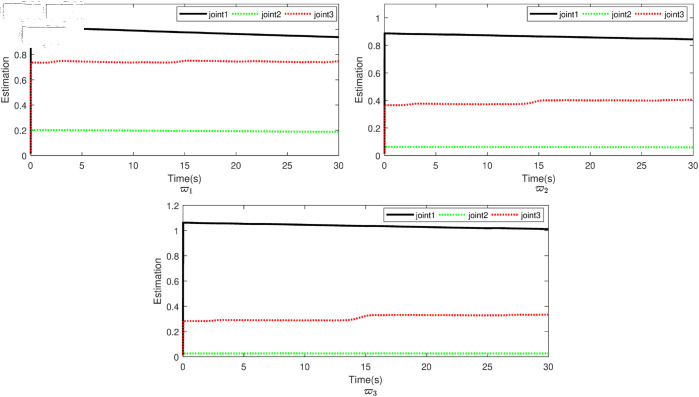
Parameter estimation under faultless operation.

**Fig 13:**
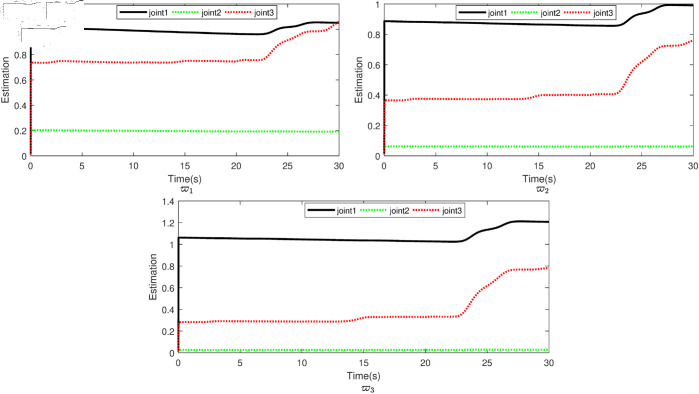
Parameter estimation under fault operation.

**Fig 14:**
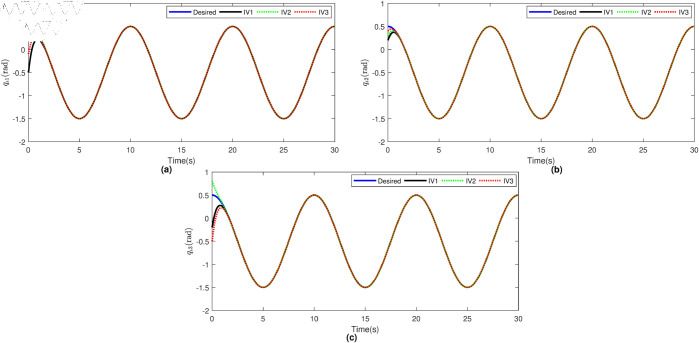
Position tracking performance.

**Fig 15:**
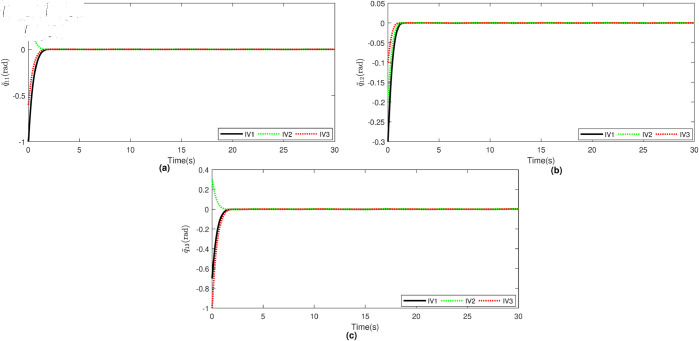
Trajectory tracking errors.

Based on the equation provided, in the simulation, we presumed an abrupt fault occurred in the first joint at t≥20s. Additionally, at t≥20s, we considered the effectiveness of the control input in the second joint to be diminished by $0.3\sin\left(t\right)u_{2}$, and we postulated an abrupt fault in the third joint at the same time. The position tracking performance and the tracking error of the system under the inputs of the CTC, ANFTSMC, Li fixed-time controller and the proposed CAFNTSMFTC are exhibited in Figs 6 and 7, respectively. Fig 6 shows that the suggested controller CAFNTSMFTC outperforms the CTC, ANFTSMC and Li fixed-time controller in terms of position tracking responsiveness in the presence of uncertainties, disturbances, and actuator faults. Additionally, it can be inferred from the overall tracking OTE= $\left\|{\tilde{q}}_{1}\right\|$ in Fig 7, that the suggested controller outperforms other controllers regarding tracking errors. The control inputs for the CTC, ANFTSMC,Li fixed-time and CAFNTSMFTC are displayed in Figs 8,9,10 and 11 respectively. Fig 11 illustrates that the proposed CAFNTSMFTC offers a continuous control input. The CTC also provides a smooth control input, as depicted in Fig 8, because it lacks the *sign* function in its design. The ANFTSMC controllers produce continuous control input, thanks to the utilization of tanh(x/ε),ε > 0 in place of sign(x) function [44]. The Li fixed-time controller also generates a continuous control input due to the application of the boundary method [45], however, as mentioned earlier, these approaches compromise system robustness and increase steady-state error. Figs 12 and 13 illustrate the adaptive estimations for ${\hat{\varpi }}_{1i},\;{\hat{\varpi }}_{2i}$ and ${\hat{\varpi }}_{3i}$, under normal and fault operation respectively, providing evidence that the suggested adaptive updating laws can attain a favorable convergence performance. As a result of the findings that have been presented in the figures, it is possible to draw the conclusion that the suggested CAFNTSMFTC offers an exceptional fault-tolerant capability and smooth control efforts. In the third phase of the simulations, we investigate the performance of the proposed control method CAFNTSMFTC under the presence of system disturbances and uncertainties, taking into account three distinct sets of initial state conditions: $IV1={\left[-0.5rad,\;0.2rad,-0.2rad\right]}^{\text{T}}$ , $IV2={\left[1rad,\;0.3rad,\;0.8rad\right]}^{\text{T}}$, $IV3={\left[-0.1rad,\;0.4rad,\;-0.5rad\right]}^{\text{T}}$. The parameters for CAFNTSMFTC are configured based on the preceding analysis. Fig 14 presents the outcomes related to position tracking, while Fig 15 displays the tracking error performance of the proposed controller across varying initial conditions. Notably, it can be observed that the convergence time remains consistent regardless of the specific initial conditions employed.

## 5 Conclusion

This work introduces a new adaptive control strategy for fixed-time trajectory tracking of a robot manipulator, even when facing challenges like actuator faults, system uncertainties, and external disturbances. To attain fixed-time control, it integrates adaptive techniques with a nonsingular fast terminal sliding surface based on the fixed-time method. This CAFNTSMFTC method, unlike existing fixed-time controls, avoids chattering and does not rely on information about lumped unknown component including including uncertainties, external disturbances and actuator faults. It also exhibits significantly faster convergence rates for system states, irrespective of their proximity to the origin, and as well as effectively addressing singularity issues. Through Lyapunov stability theory, it establishes explicit expressions for small convergence regions. Furthermore, the simulation analysis, considering uncertainties, external disturbances and actuator faults, highlights the superior tracking performance of this designed control scheme among three different control approaches.

Future research will explore the impact of additional nonlinearities such as input saturation and measurement noise on system performance. Additionally, conducting practical experiments with the proposed control approach on real robot manipulator systems is an important avenue for further investigation.
